# Nitrogen monitoring and inversion algorithms of fruit trees based on spectral remote sensing: a deep review

**DOI:** 10.3389/fpls.2024.1489151

**Published:** 2024-11-22

**Authors:** Ruiqian Xi, Yanxia Gu, Xiaoqian Zhang, Zhenhui Ren

**Affiliations:** ^1^ College of Mechanical and Electrical Engineering, Hebei Agricultural University, Baoding, China; ^2^ College of Science, Hebei Agricultural University, Baoding, China

**Keywords:** real-time monitoring, phenological period, precision management, remote sensing technology, nitrogen fertilization, fruit trees

## Abstract

Nitrogen, as one of the important elements affecting the growth and development of fruit trees, leads to slowed protein synthesis and reduced photosynthesis, resulting in yellowing of the leaves, poor tree growth, and decreased yield under nitrogen-deficient conditions. In order to minimize losses and maximize fruit yield, there is often an occurrence of excessive fertilization, soil structure degradation, and water pollution. Therefore, accurate and real-time monitoring of nitrogen content in fruit trees has become the fundamental prerequisite for precision management of orchards. Furthermore, precision orchard management is crucial for enhancing fruit quality by maintaining the optimal growth conditions necessary for trees. Moreover, it plays a vital role in safeguarding the ecological environment by mitigating the overuse of fertilizers and pesticides. With the continuous development and application of spectral remote sensing technology in agricultural monitoring and land management, this technology can provide an effective method for monitoring nitrogen content. Based on a review of relevant literature, this paper summarizes a research framework for monitoring and inversion of nitrogen content in fruit trees, which provides help for further research. Firstly, based on different remote sensing platforms, the application was discussed, on spectral remote sensing technology in the acquisition of nitrogen content in fruit trees. Secondly, the index parameters that can reflect the nitrogen content of fruit trees are summarized, which provides practical guidance for remote sensing monitoring. Additionally, the regression algorithms and application situations based on spectral data for nitrogen content were introduced. In conclusion, in response to the current issues and technological limitations, future research should focus on studying the nitrogen content characteristics of fruit trees during different phenological periods, integrating multi-type data information, and thereby improving the universality of the nitrogen content inversion model for fruit trees.

## 1 Introduction

Nitrogen is one of the essential nutrient elements required for the growth of fruit trees, playing a vital role in their development stages (Jin et al., 2022). As a component of biomass such as nucleic acid, protein and chlorophyll, nitrogen can affect the photosynthesis and chlorophyll synthesis of leaves, and then affect the growth and development of fruits, which can be directly reflected in fruit size, appearance and yield (Colpaert et al., 2021). Therefore, in pursuit of higher yields, the excessive use of nitrogen fertilizer often occurs in the process of fruit tree cultivation. Excessive nitrogen fertilizer will lead to soil acidification, destroy the stability of soil structure, stimulate the overgrowth of fruit trees and increase the risk of pests and diseases, which will reduce the yield and quality of fruit (Cui et al., 2020). At the same time, a significant portion of the applied nitrogen fertilizers leaches into water bodies rather than being absorbed by plants, leading to eutrophication. This triggers a series of environmental problems, including the disruption of the ecological balance in aquatic systems, harm to aquatic organisms, and an increase in greenhouse gas emissions (Shukla et al., 2023; Ng et al., 2024). Conversely, nitrogen deficiency can also have serious consequences for the growth of fruit trees. Nitrogen deficiency can hinder leaf photosynthesis, which is visually reflected in leaf yellowing, lightening, or shedding, ultimately leading to slowed growth or even death of the fruit tree (Gentile et al., 2022). Therefore, in the process of fruit tree cultivation, the judicious use of nitrogen fertilizer to maintain the trees in an appropriate nitrogen environment is of significant importance for increasing fruit yield, quality, and the level of refinement orchard management.

The traditional nitrogen content acquisition methods are mainly divided into organizational analysis method and soil detection method, which usually involve destructive collection of plant tissue or soil samples, and then chemical reagents for analysis and extraction are used (Cenci et al., 2021; Mohd Asaari et al., 2022). With the development of sensor technology, related portable measuring instruments have been developed and applied. These instruments are characterized by portability and operability, allowing for rapid results under non-destructive conditions. However, these instruments can only collect a single data sample at a time, failing to meet the demands of large-scale nitrogen content monitoring. Furthermore, the collection process is influenced by factors such as human operation errors and equipment sensitivity (Afonso et al., 2018). In recent years, spectral remote sensing technology, with its non-destructive, non-contact, and large area data acquisition characteristics, has been widely used in monitoring vegetation growth, diagnosing pests and diseases, and monitoring water quality and soil nutrients (Noda et al., 2021; Fu et al., 2023, 2024; Zhang et al., 2024). However, each spectral remote sensing technology has its own advantages and characteristics, leading to certain differences in agricultural applications. Therefore, selecting the most suitable practical method for orchard nitrogen content monitoring among various spectral remote sensing technologies is crucial.

The modeling methods for the inversion of nitrogen in fruit trees can be divided into statistical analysis methods, physical analysis methods, and hybrid methods (Berger et al., 2020). Statistical analysis is the most basic regression analysis method, relying on mathematical statistical theory to infer the mathematical relationship between the measured nitrogen values and spectral information, thereby obtaining the inversion results of the nitrogen status of fruit trees. It can be further divided into traditional regression methods and machine learning regression methods (Lu and He, 2019). The physical analysis methods usually involve radiation transfer theory, absorption-scattering principles, and the biochemical characteristics of leaves to capture changes between light and leaf structure, pigments, and nitrogen-containing compounds. It outputs the process of radiation absorption and scattering in the form of reflectance spectra, establishing a correlation model between nitrogen and reflectance spectra (Croft et al., 2020). However, the measurement of data such as leaf pigment, light reflectance, and compound content requires strict equipment requirements, and the availability and quality of measurement data also become limiting factors for validating physical models (Fu et al., 2020a). This model is less utilized in fruit tree nitrogen content monitoring. The hybrid methods are obtained by combining statistical analysis methods and physical analysis methods. The hybrid methods enhances the accuracy and interpretability of statistical methods, and reduces the level of expertise required for physical analysis methods (Verrelst et al., 2019). The hybrid methods can be considered as a direction for in-depth research on the construction of future inversion models for nitrogen content in fruit trees.

**Figure 1:**
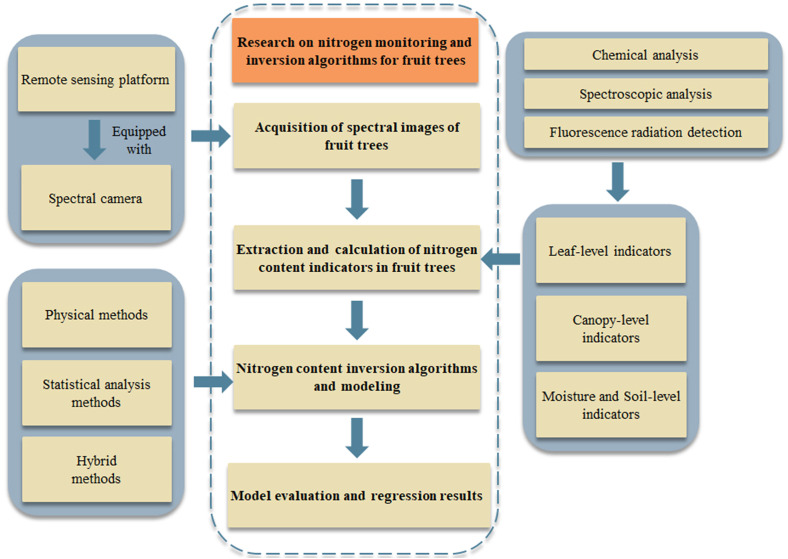
Framework for Nitrogen monitoring in fruit trees based on remote sensing technology.

In this paper, a full review of the nitrogen monitoring framework for fruit trees suitable for spectral remote sensing technology is performed. The monitoring framework is as shown in **Figure 1**. In section 2, the application of spectral remote sensing technology in nitrogen monitoring of fruit trees is introduced. Section 3 summarizes the indicators related to nitrogen content in fruit trees and provides some recommendations. In section 4, an introduction and analysis of regression algorithms in the nitrogen inversion modeling process are provided, offering references for selecting suitable algorithms and models. Section 5 summarizes and discusses the key technologies of nitrogen monitoring and inversion in fruit trees. Section 6 draws conclusions based on the preceding sections.

## 2 Remote sensing monitoring technology of nitrogen in fruit trees

Theoretically, the spectral reflectance of plant leaves is influenced by the structural composition and physiological state of leaves (Hou et al., 2024). The multispectral band generally covers the spectral range of 400-1000nm, which meets the needs of most vegetation monitoring and quantitative analysis. It can reflect the approximate spectral response characteristics of vegetation, such as the reflection peak under green wave, high reflection red edge, chlorophyll absorption peak, etc (Inoue, 2003). Hyperspectral, compared to multispectral, offers a more detailed resolution, providing continuous spectral band curves that are more sensitive to the spectral response characteristics of vegetation, reflecting a greater amount of nitrogen-related spectral information. Therefore, hyperspectral remote sensing technology can meet the more refined requirements for nitrogen monitoring (Arogoundade et al., 2023). Furthermore, with the development of remote sensing technology, various options such as satellites, aircraft, and unmanned aerial vehicles (UAV) have provided diversified modes for nitrogen monitoring in fruit trees (Feng et al., 2020).

**Figure 2:**
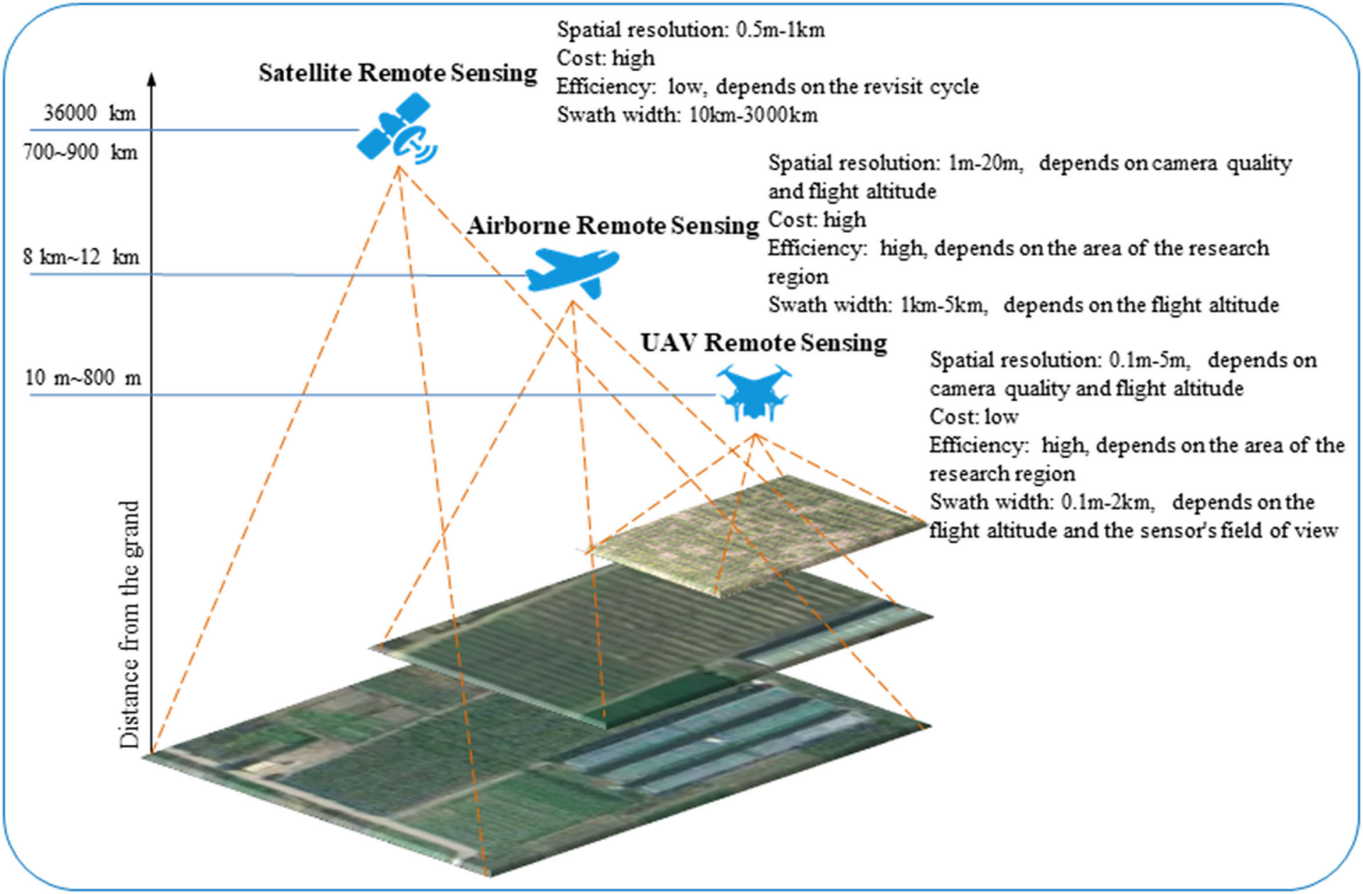
Different remote sensing platform diagrams.

**Figure 3:**
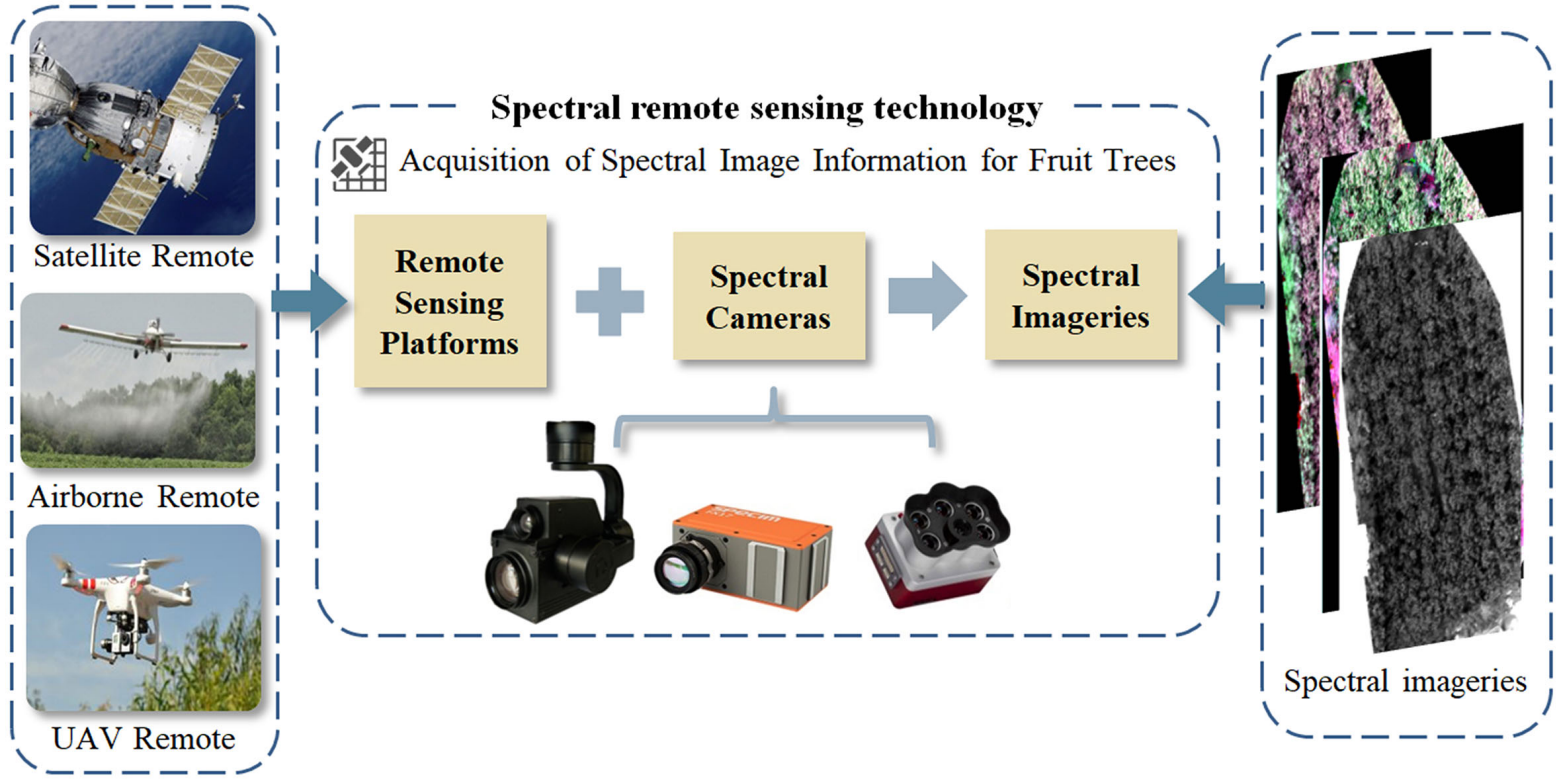
The schematic framework of the spectral remote sensing technology part.

There are differences between different remote sensing platforms in terms of space and spectral resolution, as well as cost, efficiency, and coverage, as shown in **Figure 2**. At the same time, this section presents a schematic framework of the spectral remote sensing technology part in the nitrogen monitoring process of fruit trees, as shown in **Figure 3**.

### 2.1 Satellite-based remote sensing

The satellite remote sensing platform is widely used in the field of agricultural monitoring due to its non-invasive, wide coverage, and quantifiable characteristics (Zhang et al., 2021; Zou et al., 2022). Common multispectral remote sensing satellites include the Landsat series, WorldView series, GeoEye-1 series, SPOT-7 series, Sentinel series and so on (Murugan et al., 2017; Nininahazwe et al., 2023). Beeri et al. (2005) aimed to establish a model for assessing plant nitrogen using remote sensing at a certain scale. Firstly, the leaf canopy reflectance data was obtained by ground acquisition, and the spectral characteristics and biomass model were established. Then, the multispectral satellite band information such as Landsat 5 and SPOT 5 was used to establish the model combined with the stepwise linear regression algorithm. The results provide a new approach for establishing nitrogen monitoring models (Beeri et al., 2005).

Since the year 2000, more than a dozen hyperspectral satellites have been launched globally by various countries (Verrelst et al., 2021), such as the PRISMA hyperspectral satellite from the Italy (Pepe et al., 2023), the SBG hyperspectral satellite from the United States (Lee et al., 2022), and the CHIME hyperspectral satellite from the European Space Agency (Valentini et al., 2023). It is worth noting that satellite remote sensing allows for the rapid acquisition of large-scale spectral images, making it more suitable for monitoring the biomass and soil nutrients of crops such as wheat, corn, and soybeans (Croft et al., 2019; Flynn et al., 2023). Rama Rao et al. (2008) conducted a study to investigate the utilization of hyperspectral satellite imagery for estimating total chlorophyll and nitrogen concentrations in crop leaves. They employed EO-1 Hyperion hyperspectral imagery data, supplemented by laboratory measurements from field samples of cotton and rice. Through linear regression analysis, they examined the relationship between leaf biochemical parameters and spectral reflectance, subsequently developing predictive models (Rama Rao et al., 2008). However, it has certain limitations in monitoring nitrogen content in fruit trees (Feng et al., 2019). Generally speaking, fruit trees have more complex canopy structure and higher coverage of branches and leaves. Spectral remote sensing at the satellite level may not provide sufficient spatial resolution, and image blurring may occur. Furthermore, using spectral satellites for detection may lead to data intermittency (Xia et al., 2022). Due to the phenological period characteristics of fruit trees, nitrogen or other biomass in leaves undergoes periodic changes. This can consequently impact the analysis of the overall nitrogen dynamics in fruit trees (Dannenberg et al., 2020).

### 2.2 UAV-based remote sensing

With the increasing portability of spectral cameras, they are widely used for monitoring the physiological parameters of fruit trees by being mounted on UAV (de Castro et al., 2021). Compared with satellite remote sensing platform, UAV remote sensing platform can provide higher resolution images, and its flexibility, customization and portability are easier to adapt to the environment of orchard planting (Adao et al., 2017). This approach offers greater flexibility and portability, allowing for targeted detection based on the growth cycles of fruit trees. It enables a better understanding of the dynamic changes in nitrogen levels in fruit trees and provides more precise decision support for orchard cultivation and fertilization (Ferro et al., 2023).

Osco et al. (2019) conducted research on citrus trees using an UAV equipped with a Parrot Sequoia camera (Parrot Drones SAS Inc., Paris, France). Based on the spectral information covered by the multispectral camera, 32 spectral indices were calculated. They combined these indices with the random forest (RF) regression algorithm to establish a canopy nitrogen inversion model, reducing the dependency on chemical analysis of leaf tissues. Additionally, the study utilized a variety of spectral indices as data sources for nitrogen regression analysis, providing valuable insights for nitrogen monitoring in fruit trees from an index selection perspective (Osco et al., 2019). Sun et al. (2023) used the Phantom4 Multispectral UAV (SZ DJI Technology Inc., Shenzhen, Guangdong, China) to obtain the whole multispectral image data of the orchard. Based on the noise processing of the canopy image, the spectral indices and leaf nitrogen content were further processed by regression analysis and modeling. They selected the most accurate inversion model and integrated it with a diagnosis and recommendation integrated system to provide a new technical approach for leaf nitrogen content monitoring. Notably, the study introduced fruit tree phenological period characteristics as one of the reference indices, collecting data, conducting regression analysis, and modeling the nitrogen content of apple trees during different phenological periods. The results further improve the pertinence of nitrogen monitoring of fruit trees (Sun et al., 2023). Li et al. (2022b) conducted a study on apple trees using the M600 PRO UAV (SZ DJI Technology Inc., Shenzhen, Guangdong, China) equipped with the UHD 185-Firefly (Cubert GmbH Inc, Ulm, Baden-Württemberg, Germany) to capture hyperspectral images of the tree canopies. In the process of canopy image processing, the normalized difference canopy shadow index (NDCSI) was combined on the basis of the normalized difference vegetation index (NDVI), and the shadow inside the canopy was better removed by adjusting the threshold, and the vegetation and soil were further separated in detail. Therefore, the study provides a method for further improving the accuracy of nitrogen monitoring in fruit trees by reducing shadow noise and separating soil from tree canopies (Li et al., 2022b). Similarly, Li et al. (2022a) also used apple trees as the research subject, acquiring canopy hyperspectral imagery using the M600 Pro UAV (SZ DJI Technology Inc., Shenzhen, Guangdong, China) equipped with the UHD185 (Cubert GmbH Inc, Ulm, Baden-Württemberg, Germany). In addition, based on the removal of shadow noise by NDCSI, a modified correlation coefficient method was proposed to screen the sensitive wavelengths of nitrogen content. This method can screen the characteristic bands related to nitrogen more comprehensively and accurately in the continuous spectral bands of hyperspectral, which is different from the common correlation coefficient method. In turn, the efficiency of the subsequent data analysis modeling process is improved, and the influence of redundant bands is reduced (Li et al., 2022a). Kang et al. (2023) also used apple trees as the research object, using the M300 RTK UAV (SZ DJI Technology Inc., Shenzhen, Guangdong, China) equipped with the MicroHSI 410 Shark hyperspectral camera (Corning Inc., Corning, NY, US). They used the NDVI index to separate the tree canopy from the soil and obtained spectral reflectance curves of the canopy. The canopy image obtained in this study covered the whole growth cycle of fruit trees. It provides research methods and ideas for analyzing nitrogen in fruit trees from the perspective of phenological periods, and provides technical support for refined orchard management (Kang et al., 2023).

### 2.3 Ground-based instrument methods

It is worth noting that obtaining spectral information of fruit tree canopies through remote sensing imaging methods has certain advantages and application prospects compared to traditional manual detection methods (Ju et al., 2023). However, to ensure the scientific validity and accuracy of the spectral data regression analysis process, it is still necessary to collect ground-based spectral information of fruit trees. The portable spectrometer can collect spectral information in the visible-near infrared range. The data results can be divided into imaging and non-imaging types. The imaging spectral data have spatial characteristics and can provide continuous and detailed spectral bands, so the amount of data generated is large. Non-imaging spectral data do not have spatial characteristics, only for the spectral characteristics of specific regions or data points, the amount of data generated is relatively small (Aranguren et al., 2020).

Dedeoglu (2020) used an ASD FieldSpec high-resolution spectroradiometer (Analytical Spectral Devices Inc., Boulder, CO, US) to measure the reflectance of peach leaves under three nitrogen conditions (Deficient, Sufficient, and Excess). Combined with Gaussian Mixture Discriminant Analysis, a critical nitrogen (N) content estimation model was established to monitor and distinguish the nitrogen nutrition status of peach trees (Dedeoglu, 2020). In addition, common handheld spectrometers include RapidSCAN CS-45 sensor (Holland Scientific Inc., Lincoln, Nebraska, US) (Lu et al., 2020), USB4000 (Ocean Optics Inc., Dunedin, FL, US) (Yang et al., 2019). RS-3500 (Spectral Evolution Inc., Haverhill, MA, US) (Duckena et al., 2023), MicroNIR 1700 spectrometer (Viavi Solution Inc., Milpitas, CA, US) (Castrignano et al., 2019).

In summary, for large-scale fruit tree planting areas, it is essential to combine remote sensing technology with ground-based portable measurement devices to improve the quality of spectral data. This lays the foundation for subsequent spectral index calculation and regression modeling.

## 3 Nitrogen status characteristic index of fruit trees

**Figure 4:**
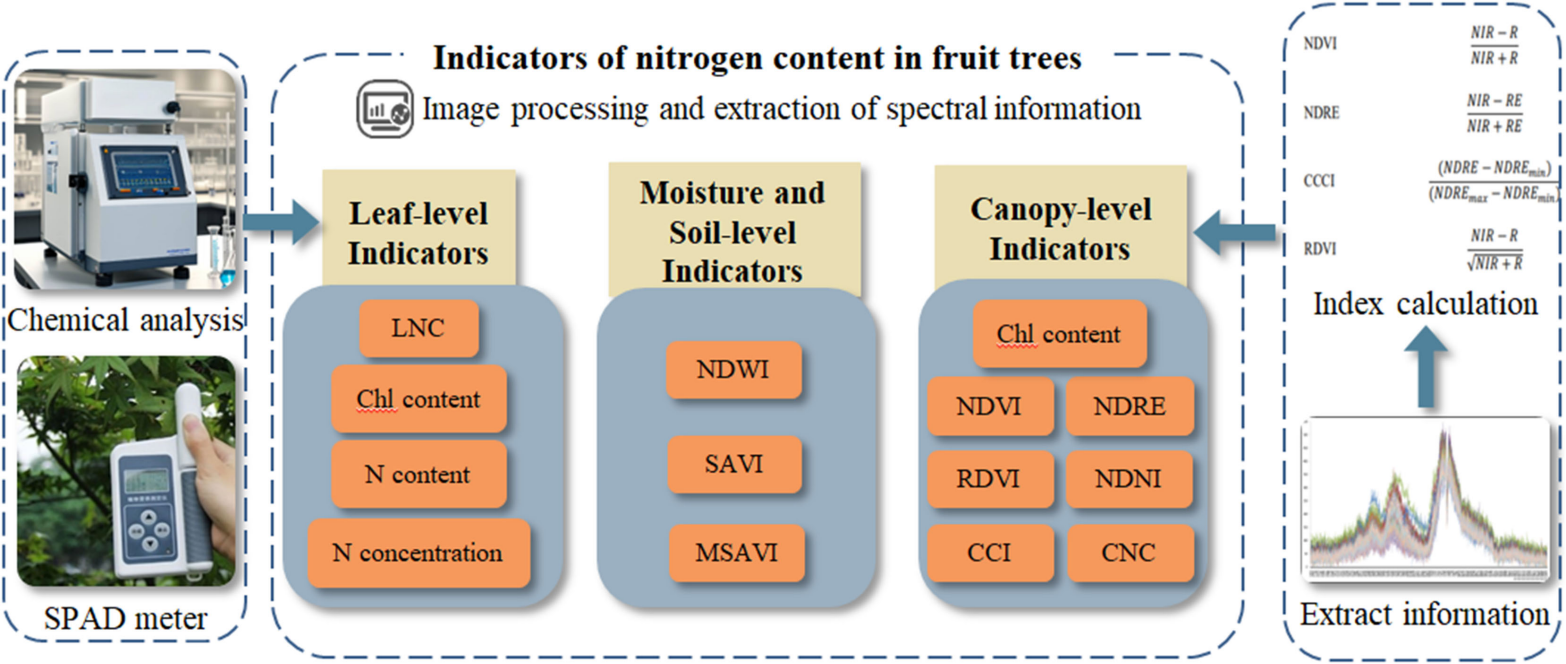
The schematic framework for acquisition and calculation of spectral indices.

The detection of nitrogen status has always been one of the important aspects of biomass monitoring in fruit trees (Berger et al., 2020). Nitrogen status can be directly reflected in the quality and yield of fruit, leaf color, plant height, plant diameter and other aspects of fruit trees (Song and Wang, 2023). It is worth noting that when differences in fruit trees are observed through these aspects, their nitrogen status has had a serious negative impact on fruit trees, even leading to the death of fruit trees and causing economic losses (Pena-Novas and Archetti, 2021). Therefore, according to the participation characteristics of nitrogen in the physiological and biochemical processes of fruit trees, the nitrogen status of fruit trees can be monitored from leaves, canopy, water content and soil parameters. This section provides a schematic framework for the characteristic indicators of nitrogen in the process of fruit tree nitrogen monitoring. The framework is as shown in **Figure 4**.

### 3.1 Leaf nitrogen index of fruit trees

First of all, from the perspective of plant biomolecules, protein is a biopolymer composed of amino acids as the main unit, and its composition is inseparable from nitrogen (Feret et al., 2021). Therefore, Johan Kjeldah proposed an auxiliary method for tracking protein content in 1883. The entire experimental process can be roughly divided into several parts: digestion, neutralization, distillation, and titration. Leaf nitrogen content (LNC), has an important impact on plant growth and development, photosynthesis, stress resistance and so on. It is a key parameter for evaluating and managing plant nitrogen nutrition (Zhang et al., 2022a). The formula calculated by Kjeldahl method is as shown in Equation 1.


(1) $$LNC\left(g\cdot kg^{-1}\right)=\frac{c\times \left(v-v_{0}\right)\times 0.014\times ts}{m\times 10^{-3}}$$

where c is the concentration of the dilute sulfuric acid solution ( mol/L ); v and $v_{0}$ are the volumes of dilute sulfuric acid used in titrating sample solution and the volume of dilute sulfur used in titrating blank, respectively ( ml ); 0.014 is the molar mass of nitrogen ( $\text{kg}\cdot {\text{mol}}^{-1}$ ); ts is the separation multiple, the ratio of constant volume to separated volume; and m is the mass of the weighted sample ( g ). Acosta et al. (2023) used persimmon leaves as experimental samples. After the analysis of Kjeldahl method, the nitrogen content of leaves was obtained, which was further used to verify the accuracy of partial least squares model based on near-infrared spectral data.

In spite of this, the use of Kjeldahl method still has certain limitations. In the course of the experiment, dangerous operations such as strong acid and high temperature are involved, and the requirements for instruments and equipment are strict. More importantly, this method is a destructive detection method, which is not conducive to the promotion and application of large areas (Acosta et al., 2023).

Furthermore, nitrogen is an important component of chlorophyll molecules, the synthesis of the porphyrin ring in chlorophyll is hindered under nitrogen stress, leading to a reduction in chlorophyll content due to a decrease in the activity of certain auxiliary enzymes. Conversely, an excess of nitrogen results in a decrease in chlorophyll content, due to the accumulation of carbohydrates in the leaves, increasing the activity of chlorophyll-degrading enzymes (Kaur et al., 2015; Wen et al., 2022). Therefore, leaf chlorophyll content is often used to reflect nitrogen status. In the process of obtaining chlorophyll content, to avoid destructive chemical analysis, chlorophyll meters have become a commonly used method in research (Abouelenien et al., 2021). Although chlorophyll meters do not directly measure the chlorophyll content of leaves, the SPAD values they provide are closely correlated with actual chlorophyll levels. By employing standardized methods for chlorophyll content measurement and mathematical analysis, a curve function model can be developed to convert SPAD values into estimated chlorophyll content (Cassol et al., 2008). SPAD-502 (Konica Minolta, Inc., Osaka, Japan) is a commonly used portable SPAD measuring instrument, and its displayed SPAD value can be expressed by Equation 2 (Naus et al., 2010).


(2) $$\begin{array}{c} SPAD=k\left[\log\left(\frac{{I^{\prime }}_{940}}{I_{940}}\right)-\log\left(\frac{{I^{\prime }}_{650}}{I_{650}}\right)\right]+c\\ \;\;\;=k\left[\log\left(T_{940}\right)-\log\left(T_{650}\right)+c\right] \end{array}$$

Where c is the compensation value adjustable in the instrument software, the quantity k (a confidential proportionality coefficient) defines the relative SPAD units, ${I^{\prime }}_{940}$ and ${I^{\prime }}_{650}$ are transmitted light intensities at respective wavelengths, $I_{940}$ and $I_{650}$ are light intensities of the light sources, and $T_{940}$ and $T_{650}$ are light transmittances through the leaf. The measurement process of the SPAD value, as a non-destructive and rapid monitoring method, exhibits good adaptability and convenience in field experimental environments (Donnelly et al., 2020). In addition, in the studies by Yue et al. (2020) and Singh and Singh (2022), the correlation between SPAD and nitrogen was also verified. Therefore, directly using SPAD value to estimate nitrogen provides a feasible method for the detection of nitrogen status of fruit trees (Yue et al., 2020; Singh and Singh, 2022). However, the measurement process of the portable chlorophyll measuring instrument can only collect a single point, and the data quality is greatly affected by the leaf chlorophyll distribution and the measurement position (Tan et al., 2021).

The chlorophyll fluorescence measurement method also serves as a non-destructive means of detecting chlorophyll content (Padhi et al., 2021). **Table 1** summarizes the research situation of portable chlorophyll fluorescence measuring instrument in chlorophyll content detection. It is worth noting that chlorophyll fluorescence parameters are susceptible to environmental factors such as light intensity, leaf moisture, and temperature. Additionally, with changes in the phenological periods of fruit trees, both chlorophyll content and fluorescence parameters undergo dynamic changes. Relying solely on chlorophyll parameters cannot comprehensively capture the nitrogen variation trend throughout the entire phenological periods of fruit trees (Kasampalis et al., 2021; Lin et al., 2023).

**Table 1:** Summary of research based on portable chlorophyll fluorescence measuring instruments.

Device Model	Measurement Parameters	Results	Ref.
OS30P+ (Opti-Sciences Inc., Hudson, NH, US)	$F_{V}/F_{m}$ (the potential maximum photochemical efficiency of PSII)	1. There is a positive, saturated relationship between leaf %N and chlorophyll fluorescence measurement indices. 2. Deciduous shrub species significantly increased leaf %N under high nutrient addition levels.	(Prager et al., 2020)
PAM-2100 (Heinz Walz GMBH Inc., Nuremberg, Bavaria Germany)	$F_{V}/F_{0}$ (the maximum light energy conversion potential of PSII) $F_{V}/F_{m}$ (the potential maximum photochemical efficiency of PSII) $F_{V}^{'}/F_{m}^{'}$ (the maximum photochemical efficiency of PSII)	1. The chlorophyll fluorescence parameters $F_{V}/F_{0}$ and $F_{V}/F_{m}$ increase with increasing nitrogen fertilizer application levels. 2. The vertical distribution characteristics of leaves have certain effects on chlorophyll fluorescence parameters.	(Ding et al., 2022)
MultispeQ (PhotosynQ Inc., East Lansing USA)	$q^{P}$ (photochemical quenching coefficient) $F_{V}/F_{0}$ (the maximum light energy conversion potential of PSII) $F_{V}/F_{m}$ (the potential maximum photochemical efficiency of PSII) $F_{V}^{'}/F_{m}^{'}$ (the maximum photochemical efficiency of PSII) NPQ (non-photochemical quenching coefficient)	1. The chlorophyll fluorescence parameters: $F_{V}^{'}/F_{m}^{'}$ , $F_{V}/F_{m}$ , and NPQ show significant positive correlations with leaf nitrogen content at various growth stages. 2. During the growth stages, the optimal response of chlorophyll fluorescence parameters to nitrogen content retrieval occurs during the bud stage ( $R^{2}$ =0.745) and flowering stage ( $R^{2}$ =0.709).	(Lin et al., 2023)
Handy-PEA (Hansatech Inc., Norfolk, Kings Lynn UK)	$F_{V}/F_{m}$ (the potential maximum photochemical efficiency of PSII) PI (the performance index on absorption basis)	1. The decrease in $F_{V}/F_{m}$ and PI indices with the progression of drought stress reflects the impact of drought on photosynthesis and chlorophyll content.	(Boussadia et al., 2023)

In summary, as one of the primary sites for plant metabolism and development, leaves play a crucial role. The nutritional status of fruit trees can be evaluated by measuring the nitrogen content in leaves, but the nitrogen in leaves is easily affected by growth stage and canopy structure (Feng et al., 2023). Canopy is the main interface between plants and the external environment, which can more comprehensively reflect the nitrogen operation status of the whole fruit tree. At the same time, spectral remote sensing technology can provide a fast and non-destructive nitrogen monitoring ability for canopy nitrogen monitoring (Wu et al., 2021).

### 3.2 Nitrogen index of fruit tree canopy

Compared to chlorophyll content, canopy spectral features can provide richer information, with stronger resistance to interference. At the same time, some sensitive bands have strong correlation with nitrogen, and the calculated canopy spectral characteristic index has certain universality (Chen et al., 2022).

The vegetation indices (VIs) primarily involve obtaining electromagnetic wave reflection information from the vegetation canopy through passive sensors. According to the spectral characteristics of vegetation differences, the spectral vegetation index is obtained by mathematical operation of the reflectivity of different bands, and then used to monitor or evaluate the physiological and biochemical conditions of vegetation (Radocaj et al., 2023).

The NDVI is calculated through a normalization process using the reflectance of red and near infrared bands. The calculation formula is as shown in Equation 3. It is widely applied in measuring vegetation coverage, calculating tree diameters, and monitoring the growth status of fruit trees, among other uses (Fernandez-Figueroa et al., 2022).


(3) $$NDVI=\frac{NIR-R}{NIR+R}$$

The NDVI values range from 0 to 1, where values closer to 1 indicate higher vegetation coverage in the region, and values closer to 0 indicate lower vegetation coverage, even for areas with relatively low vegetation cover. Furthermore, by replacing the red band with the red-edge band in the NDVI, the normalized difference red-edge (NDRE) index is obtained. The calculation formula is as shown in Equation 4.This is because the correlation between nitrogen content and red-edge reflectance is more pronounced (Bonfil, 2017).


(4) $$NDRE=\frac{NIR-RE}{NIR+RE}$$

Perry et al. (2018) used UAV to monitor nitrogen levels by obtaining the reflectance of the red pear canopy and leaves. The results of their regression model validate that the NDRE index is more effective than the NDVI index in monitoring nitrogen content (Perry et al., 2018). Additionally, the paper introduced another vegetation index that is more sensitive to nitrogen: canopy chlorophyll content index (CCCI). The calculation formula is as shown in Equation 5.


(5) $$CCCI=\frac{\left(NDRE-NDRE_{min}\right)}{\left(NDRE_{max}-NDRE_{min}\right)}$$

Where $NDRE_{min}$ and $NDRE_{max}$ depend on the NDVI value of the response. This index is calculated based on the NDRE index and is more sensitive to canopy nitrogen. However, due to the limitations of fruit tree canopy structure, it exhibits some instability in denser canopy structures. It is more commonly applied in the retrieval of canopy nitrogen for cereal crops (Darra et al., 2021; Chen et al., 2023).

In 1995, Roujean and Breon proposed the renormalized difference vegetation index (RDVI), which further optimized the NDVI (Roujean and Breon, 1995). The calculation formula is as shown in Equation 6.


(6) $$RDVI=\frac{NIR-R}{\sqrt{NIR+R}}$$

The square root term is introduced into the denominator of the formula to improve the linear correlation of vegetation biomass and reduce the influence of mixed background such as soil (Xue and Su, 2017). Based on the M600 Pro UAV (SZ DJI Technology Inc., Shenzhen, Guangdong, China), equipped with a Parrot Sequoia multispectral camera (Parrot Drones SAS Inc., Paris, France), Zhang et al. (2023) obtained multispectral images of apple tree canopy, aiming to retrieve apple tree canopy nitrogen content (CNC) by the vegetation index VIs calculated by spectral reflectance. The results show that the calculated RDVI value has a good correlation with CNC (Zhang et al., 2023).

Under the shortwave infrared bands, nitrogen also exhibits regular spectral response characteristics. According to the research by Serrano et al., 1510 nm was identified as the optimal wavelength for nitrogen prediction, and 1680 nm was used as the reference wavelength. They introduced the normalized difference nitrogen index (NDNI) to invert canopy nitrogen (Serrano et al., 2002). The calculation formula is as shown in Equation 7.


(7) $$NDNI=\frac{\text{log}\left(1/R_{1510}\right)-log\left(1/R_{1680}\right)}{\text{log}\left(1/R_{1510}\right)+log\left(1/R_{1680}\right)}$$

Where $R_{1510}$ and $R_{1680}$ represent the reflectivity at 1510 nm and 1680 nm, respectively. Wang et al. (2016) calculated the NDNI value based on the acquired hyperspectral satellite remote sensing data to invert the nitrogen content of the forest canopy. The research results demonstrate that NDNI values can accurately predict leaf nitrogen content. Additionally, the research results also indicate that the estimation accuracy of NDNI is influenced by phenological periods and soil background factors (Wang et al., 2016).

### 3.3 The nitrogen index in terms of water and soil

There is a certain correlation between leaf moisture content (LMC) and nitrogen content. In the internal structure of plants, water content will affect the absorption and transport efficiency of nitrogen (Mwinuka et al., 2022). In terms of spectral response, the water content on the leaf surface can affect the amount of light reflected from the leaf surface. The color and light absorption differences measured by the reflectance sensors can indirectly reflect the nitrogen content. Normalized difference water index (NDWI) is the most commonly used index to measure LMC, which can indirectly reflect the nitrogen content of leaves (Marcone et al., 2024). The calculation formula is as shown in Equation 8.


(8) $$NDWI=\frac{NIR-SWIR}{NIR+SWIR}$$

Huete (1988) proved that the NDVI index has limitations in the spectral response of the soil background. Therefore, the soil-adjusted vegetation index (SAVI) was proposed (Huete, 1988).

Compared with the NDVI index, a simple model was established to fully describe the soil-vegetation system (Bannari et al., 1995; Noori and Panda, 2016). The calculation formula is as shown in Equation 9.


(9) $$SAVI=\left[\frac{\left(NIR-R\right)}{\left(NIR+R+l\right)}\right]\times \left(1+L\right)$$

Where L is the soil adjustment factor, and the value of L = 0.5 can achieve the best adjustment, that is, to minimize the secondary scattering effect of the soil background reflected radiation transmitted by the canopy. If the value of L is zero ( L = 0), SAVI is equal to NDVI. Wang and Wei (2016) used the characteristics of SAVI to optimize the NDNI index. Firstly, the red band in SAVI was replaced by 1510 nm band. Secondly, the original NDNI is divided by the revised SAVI (Wang and Wei, 2016). The calculation formula is as shown in Equation 10.


(10) $$\frac{NDNI}{SAVI_{1510}}=\frac{\left(log\left(1/R_{1510}\right)-log\left(1/R_{1680}\right))/(log\left(1/R_{1510}\right)+log\left(1/R_{1680}\right)\right)}{\left(1+L\right)\left(R_{800}-R_{1510}\right)/\left(R_{800}+R_{1510}+L\right)}$$

Where L is a self-adjusting variable related to background adjustment effect. In the article, it is pointed out that the optimized NDNI index can reduce the interference of soil background noise and improvthe accuracy of canopy nitrogen monitoring when the spectral image background may be water, too wet soil or relatively dry land.

As researchers further investigate the soil background sensitivity of VIs, Rondeaux et al. (1996) conducted a comparative analysis of the SAVI, the transformed soil-adjusted vegetation index (TSAVI), and the modified soil-adjusted vegetation index (MSAVI), among which MSAVI exhibits good stability and sensitivity. MSAVI considers the adjustment factor L in the SAVI index as a function that varies inversely with vegetation amount, minimizing the impact of bare soil on SAVI (Rondeaux et al., 1996). The calculation formula is as shown in Equation 11.


(11) $$MSAVI=\frac{2NIR+1-\sqrt{{\left(2NIR+1\right)}^{2}-8\left(NIR-R\right)}}{2}$$

Roma et al. (2023), in their study on the inversion of nitrogen in oil canopies, discovered that the MSAVI was capable of representing more nitrogen information compared to the NDVI and NDRE (Roma et al., 2023). It is worth noting that while leaf moisture content and soil regulation indices can monitor and invert the nitrogen content of fruit tree canopies, in most research processes, the NDWI is used to monitor the degree of water stress, while indices such as SAVI, TSAVI, and MSAVI are used to assess the nutrient status of orchard soils (Arevalo-Ramirez et al., 2020; Zhen et al., 2021; Zongfan et al., 2022).

In summary, VIs have become one of the important methods in precision agriculture and agricultural monitoring. They offer a non-destructive and efficient approach to monitoring various aspects of plants, such as chlorophyll and nitrogen content, leaf area, and moisture status, based on different index calculation methods. On the other hand, with the continuous advancement of spectral remote sensing technology, the selection and combination of multiple spectral bands have also opened new possibilities for the study of vegetation indices. **Table 2** summarizes the information on the vegetation indices mentioned in the text above.

**Table 2:** Summary of Vegetation Indices.

Index	Formula	Wavelengths	Application
NDVI	$\frac{NIR-R}{NIR+R}$	(600-1000) nm	One of the most commonly used indices, it is widely applied to measure fractional vegetation cover, distinguish between vegetation types, and assess land use conditions.
NDRE	$\frac{NIR-RE}{NIR+RE}$	(700-1000) nm	Compared to NDVI, this index is more sensitive to nitrogen monitoring and is widely used for assessing and monitoring plant health under varying levels of stress.
CCCI	$\frac{\left(NDRE-NDRE_{min}\right)}{\left(NDRE_{max}-NDRE_{min}\right)}$	(700-1000) nm	The sensitivity to nitrogen has been enhanced based on NDRE; however, due to limitations imposed by the canopy structure, it is primarily utilized for the health monitoring and assessment of field crops.
RDVI	$\frac{NIR-R}{\sqrt{NIR+R}}$	(600-1000) nm	This index, which optimizes the NDVI, is widely used for monitoring plant growth conditions under various soil types.
NDNI	$\frac{log\left(1/R_{1510}\right)-log\left(1/R_{1680}\right)}{log\left(1/R_{1510}\right)+log\left(1/R_{1680}\right)}$	1510nm&1680nm	The index, calculated in the Short Wave Infrared (SWIR) band, is commonly used to monitor and evaluate plant growth conditions in regions with notably complex canopy structures.
NDWI	$\frac{NIR-SWIR}{NIR+SWRI}$	(700-2500)nm	By assessing nitrogen levels based on leaf water content, this index is more frequently applied to monitor and evaluate plant growth under different water stress scenarios.
SAVI	$\left[\frac{\left(NIR-R\right)}{\left(NIR+R+l\right)}\right]\times \left(1+L\right)$	(600-1000) nm	This index, which enhances NDVI with an added soil adjustment factor (*L*), is widely utilized for monitoring nitrogen levels in regions where vegetation cover is sparse.
MSAVI	$\frac{2NIR+1-\sqrt{{\left(2NIR+1\right)}^{2}-8\left(NIR-R\right)}}{2}$	(600-1000) nm	Compared to SAVI, this index is more commonly used for nitrogen monitoring in areas with higher vegetation cover.

## 4 Nitrogen inversion method of fruit trees based on spectral data

**Figure 5:**
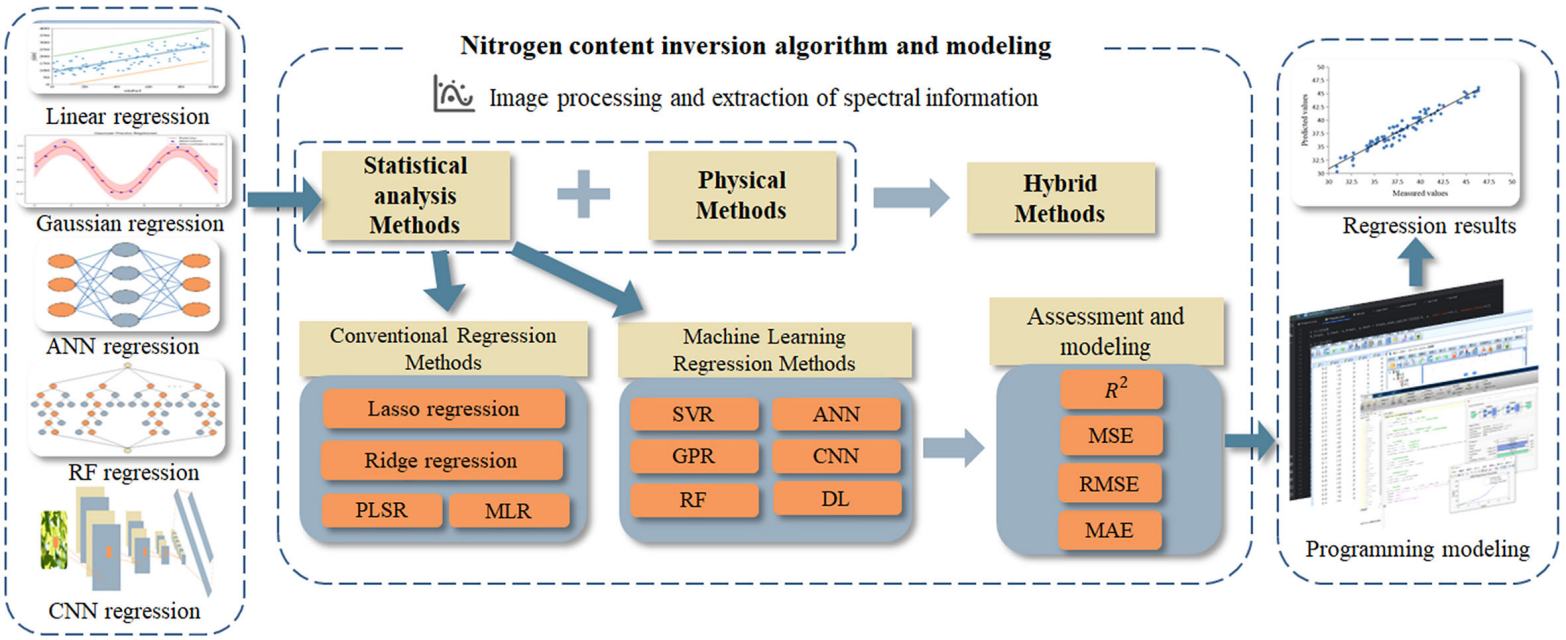
The schematic framework for nitrogen content retrieval and regression modeling.

In the process of establishing a model for nitrogen content inversion in fruit trees, it is necessary to derive the relationship between nitrogen and relevant indicator parameters through a regression inversion process, aiming to reconstruct the actual nitrogen content of the fruit trees as accurately as possible. Therefore, it is necessary to select the appropriate modeling method according to the characteristics of the data set and the research needs to maximize the accuracy of the model prediction. This section gives a schematic framework for the nitrogen inversion algorithm and modeling part of the nitrogen monitoring process of fruit trees. The framework is as shown in **Figure 5**.

### 4.1 Conventional regression methods

The traditional regression methods are based on simple mathematical and statistical principles, focusing on establishing a linear correspondence between one or more independent variables (explanatory variables) and a dependent variable (response variable). The least square regression (LSR), as a classic linear regression algorithm, primarily aims to construct the optimal function expression by minimizing the sum of squared errors in the data (Darvishzadeh et al., 2008). Its objective is to establish a linear mathematical model as Equation 12.


(12) Y=βX+ϵ

Where Y is the mean centered vector of the dependent variable, X is the mean centered matrix of the independent variable, β is the coefficient matrix, and ϵ is the residual matrix. The partial least squares regression (PLSR) is similar to it. PLSR performs better in handling multicollinearity among independent variables, even though both algorithms calculate model parameters by minimizing error values. It involves screening and eliminating a large number of collinear independent variables and retaining some unrelated latent variable factors to establish the model by maximizing the covariance between the variable data (Helland et al., 2018).

For single-variable regression models, as phenological period characteristics and multiple spectral indices are introduced, a single variable is unable to fully describe the complex relationship between spectral information and nitrogen (Brinkhoff et al., 2019). Therefore, multiple linear regression (MLR) is a suitable choice. MLR can establish a linear relationship model between the dependent variable (response variable) and multiple independent variables (explanatory variables) (Shao et al., 2021). The model is represented as shown in Equation 13.


(13) $$y=\beta_{0}+\beta_{1}x_{1}+\beta_{2}x_{2}+\cdots \beta_{i}x_{i}+\epsilon$$

Where $\beta_{i}$ is the regression coefficient of variable i , and ϵ is the deviation constant. The key to influencing the multiple linear regression model is whether the parameters in the training sets are related to nitrogen. Therefore, the stepwise multiple regression (SMR) is further improved, and the highest correlation group is gradually selected from the potential independent variables to construct the regression model, so as to simplify the model without changing the prediction accuracy (Jin et al., 2020a).

Another key factor that influences the MLR model is the phenomenon of overfitting. The introduction of regularization algorithms can effectively solve this problem and reduce the potential collinearity between variables (Tak and Inan, 2022). There are two common regularization algorithms: lasso regression and ridge regression. Lasso regression, also known as L1 regularization, adds a penalty term of the sum of the absolute values of the model parameters to the loss function (Ng and Newton, 2022), as shown in Equation 14.


(14) $$\lambda \sum_{i=1}^{n}\left|\beta_{i}\right|$$

Where λ is the regularization parameter, and $\beta_{i}$ is the regression coefficient. Ridge regression, also known as L2 regularization, adds a penalty term to the sum of squares of model parameters in the loss function (Zhou et al., 2021), as shown in Equation 15.


(15) $$\lambda \sum_{i=1}^{n}\beta_{i}^{\;2}$$

Where λ is the regularization parameter and $\beta_{i}$ is the regression coefficient. Ridge regression, compared to lasso regression, is more effective in addressing collinearity issues (Kerckhoffs et al., 2019). In a study by Osco et al. (2020), based on the collected spectral parameters of citrus leaves, lasso regression, ridge regression, and machine learning methods were used to estimate the correlation between spectral features and nitrogen. The results indicate that both lasso regression and ridge regression algorithms have some predictive capability, but their effectiveness is far more inferior to that of machine learning methods (Osco et al., 2020).

In conclusion, linear models still have many advantages, such as simplicity, high computational efficiency, and wide applicability. However, it is equally important to consider the characteristics of the dataset and the requirements of the research application. The simplistic assumption of a linear relationship between variables can lead to an oversimplification of the model, failing to accurately fit the actual trends in the data (Ma et al., 2024). Therefore, machine learning methods can achieve better results when dealing with non-linear relationships between variables (Ma et al., 2022a).

### 4.2 Machine learning regression method

Spectral remote sensing technology provides non-linear and complex spectral information. However, machine learning methods can screen and extract valuable eigenvalues from a large amount of data, and are used in complex data regression analysis tasks (Sabzi et al., 2022). At the same time, its precise predictive ability for data becomes particularly important in the environmental conditions of fruit tree growth. With the increase in the variety and quantity of training data, the performance of machine learning regression models also improves, and their versatility and scalability will further meet the requirements of agricultural remote sensing information analysis (Virnodkar et al., 2020).

#### 4.2.1 Machine learning regression method based on kernel function

The kernel function significantly impacts the performance of regression, as it determines the shape of the regression function and provides a method for high-dimensional space mapping: it maps the original input space to a high-dimensional feature space, where the linear relationships in the data are more easily fitted (Pourbahrami et al., 2023). Common kernel functions can be classified into local and global kernel functions, including linear kernel, polynomial kernel, and radial basis function (RBF) (Wang et al., 2020). The following function expressions as shown in the **Table 3**.

**Table 3:** Summary of kernel function expressions.

Kernel Function	Expression	Notes
Linear Kernel	$K\left(x,x_{i}\right)=x_{i}^{\;T}x_{j}$	1. $x_{i}$ and $x_{j}\;$ are date points. 2. The linear kernel does not change nonlinearly, and directly calculates the dot product of the vector.
Polynomial Kernel	$K\left(x,x_{i}\right)={\left(x_{i}^{\;T}x_{j}\right)}^{d}$	1. $x_{i}$ and $x_{j}\;$ are date points, and d is coefficient of polynomial. 2. The feature can be mapped to a high-dimensional space, and the dimension is proportional to the value of d, which is prone to overfitting when it is too high.
Radial Basis Function (RBF)	$K\left(x,x_{i}\right)=exp\left(-\frac{{\left\|x_{i}-x_{j}\right\|}^{2}}{2\sigma^{2}}\right)$	1. $x_{i}$ and $x_{j}\;$ are date points, and σ is width of the RBF kernel. 2. The mapping dimension is higher, and the generalization ability of the model is affected by the σ value.

In the process of using spectral remote sensing data for fruit tree biomass monitoring, support vector machine (SVM) serves as a non-parametric supervised statistical learning algorithm applied to pattern recognition, classification, and regression tasks. Unlike the fixed functional relationships assumed in traditional regression techniques, SVM does not make assumptions about the underlying data distribution. Therefore, training with a small number of samples does not affect the accuracy of the model (Termin et al., 2023; Tian et al., 2023). When applied to regression analysis, this algorithm can also be represented as support vector regression (SVR), iteratively mapping the dataset to different categories in a multi-dimensional space until finding the maximum margin hyperplane (Wang et al., 2021). The model is represented as shown in Equation 16.


(16) $$f\left(x\right)=\sum_{i=1}^{n}\left(a_{i}-a_{i}^{*}\right)K\left(x,x_{i}\right)+b=\sum_{i=1}^{n}w_{i}K\left(x,x_{i}\right)+b$$

Where $\;K\left(x,x_{i}\right)$ is the kernel function, $a_{i}$ and $a_{i}^{*}$ are Lagrange multipliers, b is an adjustable parameter, and n is the number of sample sets. The choice of kernel function depends on the data characteristics and the specific needs of the problem.

The linear kernel performs well when the data is approximately linear. The polynomial kernel provides polynomial mappings of different degrees, which can handle more complex non-linear relationships. The RBF is very popular in many practical applications due to its flexibility (Torres et al., 2019). Li et al. (2022a) based on hyperspectral images of apple tree canopies, after selecting nitrogen-sensitive wavelengths and constructing spectral feature parameters, built a canopy nitrogen inversion model based on SVM (Li et al., 2022a).

Gaussian process regression (GPR) is a non-parametric Bayesian regression algorithm (Van Wittenberghe et al., 2014). Similar to the SVM algorithm, GPR does not assume the distribution of the data set either, but adjusts the parameters by observing the mean and covariance (Zhen-qi et al., 2023). Therefore, under the condition of weak prior knowledge, GPR can be used as one of the effective methods for monitoring nitrogen content (Fu et al., 2020b). Both GPR and SVR belong to the regression algorithms based on kernel function. Therefore, the performance of GPR also depends on the selected kernel function (Peng and Li, 2014). Gao et al. (2023) constructed a leaf nitrogen content inversion model based on hyperspectral information from citrus leaves and utilized various spectral denoising algorithms. The regression algorithms employed include PLSR, SVR, and GPR. The results indicate that under different data-denoising algorithm conditions, the best nitrogen response model is the GPR model based on the RBF kernel. The introduction of the RBF kernel reduces the risk of overfitting, adds smoothness, and increases sensitivity to the non-linear relationships between variables (Gao et al., 2023).

#### 4.2.2 Machine learning regression method based on ensemble learning methods

Ensemble learning is a method of constructing models by combining multiple base learners or algorithms. Its main idea is to use an ensemble to improve the model’s generalization ability across various data aspects, reducing the bias and variance of a single model through voting or averaging, thereby obtaining more accurate prediction results. Common ensemble learning methods include bagging, boosting, and stacking (Rooney et al., 2006). Bagging is an algorithm that divides the original data set into multiple subsets and combines them by bootstrap sampling method, and finally integrates the prediction results by voting or averaging (Dong et al., 2020).

The RF is an ensemble learning method based on decision tree, which introduces randomness on the basis of bagging algorithm (Ganaie et al., 2022).The algorithm based on decision tree is a common form of tree-based algorithm. Different from the kernel-based algorithm, the tree-based regression algorithm uses a tree structure when segmenting data regions, and uses leaf nodes to represent predicted values (Uddin and Lu, 2024). Therefore, it has better results in dealing with large-scale and non-linear data. However, overfitting is a common issue in the regression process of random forest, making it crucial to adjust parameters such as the number of regression trees, depth, and input variables in the model (Jin et al., 2020b). The complexity of a RF model is jointly determined by the number of trees and their depth. Increasing these parameters enhances the model’s complexity and can improve its ability to address intricate problems. Setting a higher minimum sample size for leaf nodes promotes a more conservative model, thereby reducing the risk of overfitting. However, it is important to note that the interpretability of RF regression models is generally limited, and their performance is heavily dependent on the parameter choices made during model construction. Shah et al. (2019) found that when the vegetation index is used as the input feature of random forest, the accuracy of the model is improved, which is consistent with the research results of Osco et al. (2019) (Osco et al., 2019; Shah et al., 2019). Zhao et al. (2024) utilized apple canopy hyperspectral images to extract 14 VIs related to nitrogen. They employed three algorithms, namely PLSR, ridge regression, and RF, to establish a predictive model for the LNC of the tree. The results indicate that the inversion model based on RF demonstrated the highest accuracy. In addition, the concept of phenological periods was introduced in this paper, and the LNC under different phenological periods conditions of fruit trees was predicted and analyzed (the accuracy of the model was the highest in the fruit expansion period), which further provided technical support for precise nitrogen application management in orchards and provided certain scientific basis for fruit tree growth (Zhao et al., 2024).

Boosting minimizes the residual of the previous model by gradually training the model, and then gradually improves the performance of the overall model. In the training process, each basic model will adjust the weight of the sample according to the prediction error of the previous model. Adaptive boosting (Adaboost), gradient boosting machine (GBM), extreme gradient boosting (XGBoost) and light gradient boosting machine (LightGBM) are common forms of boosting algorithm (Kleinberg, 2000). Jafarzadeh et al. (2021) conducted a study based on three actual datasets including multispectral, hyperspectral, and polarimetric synthetic aperture radar (PolSAR) data, to investigate the classification performance of different ensemble learning algorithms such as RF, bagging, and boosting. Their research findings indicated that XGBoost outperformed other algorithms in terms of effectiveness (Jafarzadeh et al., 2021).

XGBoost was proposed by Chen and Guestrin in 2016 (Chen and Guestrin, 2016). The XGBoost algorithm improved upon the gradient boosting decision tree (GBDT) algorithm. It determined the form of the loss function through second-order Taylor expansion and introduced a regularization term into the loss function. As the regularization weight increases, the model becomes more conservative, which helps to mitigate overfitting. Additionally, it adopted a tree growth strategy, allowing for adjustments in the tree’s depth and the minimum weight of leaf nodes. Higher values for both parameters result in a more complex model, improving its performance on regression tasks. As a result, the XGBoost algorithm is more accurate than the GBDT algorithm and is less prone to overfitting (Ye et al., 2021). Canting Zhang et al. (2023) constructed a model for the inversion of CNC in apple orchards using the XGBoost algorithm based on the fusion of multispectral and hyperspectral data. The model, incorporating canopy abundance parameters, demonstrated good accuracy and stability (Zhang et al., 2023).

Stacking model was proposed by Wolpert in 1992 and has been widely used in the field of machine learning (Wolpert, 1992). As an ensemble learning method, it achieves the prediction results of integrated multiple basic learners or models as new eigenvalues, and then merges the feature matrix to obtain a meta-model to process the new eigenvalues and obtain the final prediction results. This method leverages the strengths of individual base models or learners to enhance the overall predictive accuracy and generalization capability of the model (Sesmero et al., 2015). Shaomin Chen et al. (2022) established a stacked ensemble extreme learning machine model based on the hyperspectral reflectance of apple tree canopy to invert the nitrogen content in leaves (Chen et al., 2022).

#### 4.2.3 Machine learning regression method based on neural network

Neural network is a computational model inspired by the biological nervous system. It is designed to recognize, classify, and predict based on the complex relationships between data. It takes reference from the information transmission and processing processes between neurons in the human brain (Sridhar et al., 1996). In the regression task, the purpose of this method is to capture the mapping relationship between input features and output variables, and to predict the continuous output of a given input data. Artificial neural network (ANN) is a mathematical model that resembles the structure and function of biological neural networks, consisting of a large number of interconnected neurons for information processing and transmission (Sun et al., 2022). It has a multi-layer structure, with input and output layers for receiving and outputting information, while data feature extraction and processing occur in the hidden layers (Tracey et al., 2011). By adjusting the weights of each neuron to reduce errors, ANN enhances its data processing capabilities, by enabling it to adapt to the complex nonlinear relationships between canopy spectral features and biophysical parameters (Yan et al., 2023). Generally speaking, the number of hidden layers in the network is uncertain, which needs to be determined according to the characteristics of the data set (Wang et al., 2009). Noguera et al. (2021) used multispectral information from five bands, including blue, green, red, red edge, and near-infrared, extracted from canopy images of olive orchards captured by drones as the basis for building a relatively simple network structure. This structure includes one hidden layer with one neuron, five spectral information input nodes, and one LNC output layer. The levenberg-marquardt algorithm was chosen as the training algorithm based on the number of data set divisions and computational complexity. The results indicate that the ANN model shows a good predictive response to LNC (Noguera et al., 2021). Back propagation neural network (BPNN), as a special type of ANN, is improved on the basis of multilayer Perceptron (MLP). It continues the multi-level network structure and adds the back propagation algorithm (Sarkar et al., 2023). By calculating the gradient of the loss function, the connection weight in the network is adjusted to minimize the error between the predicted output and the actual output. Its characteristic is that the error propagates from the output layer to the first hidden layer during the training process, and gradually optimizes the network (Li et al., 2021). The back propagation algorithm is added to further improve the fitting ability of the BPNN network for nonlinear data (Ma et al., 2022b). Li et al. (2022b) constructed a BPNN model for monitoring apple CNC based on the hyperspectral information of apple canopies. The model includes a hidden layer with four neurons and utilizes the levenberg-marquardt as the training algorithm. In comparison to ANN, BPNN demonstrates an improved precision in nitrogen response modeling (Li et al., 2022b).

Quantitative assessment of plant biomass is not limited to extracting spectral data from leaves. Collecting leaf images, including direct acquisition of color, shape, and texture information, can also serve as indicators for biometric measurements (Schreiber et al., 2022). As an important technical branch of machine learning, deep learning has been continuously promoted and applied in agriculture. It can autonomously learn features from raw data, and the characteristics of unsupervised learning are also different from traditional machine learning methods (Nan et al., 2020).

Based on the neural network architecture, convolutional neural network (CNN) introduces a specific hierarchical structure of convolution layer, pooling layer and fully connected layer. In the convolutional layers, the convolution kernels extract feature maps from the original images, which are then compressed through the pooling layers. This process of convolution and pooling is repeated multiple times, and upon reaching the fully connected layer, the network learns the feature mappings and produces the target results based on the nature of the task (prediction or classification) (Okyere et al., 2023). Baesso et al. (2023) captured digital RGB images of bean canopies using a camera, and classified the images based on nitrogen content. By training a CNN model, they developed four nitrogen content classifiers, demonstrating the significant potential of using RGB images and deep learning techniques for nitrogen status monitoring (Baesso et al., 2023). It is worth noting that in the monitoring of canopy nitrogen status of fruit trees, there are relatively few studies using CNN and RGB methods. This paper believes that it may be limited by factors such as complex canopy structure and difficulty in image calibration and training.

The machine learning method can effectively invert the physiological and biochemical structural characteristics of plants and reveal the dynamic changes of biomass caused by the environment (Chlingaryan et al., 2018). With the continuous deepening of research on machine learning methods, various aspects of the data set quality, model parameters, and the structure of the regression function of machine learning models have been optimized and improved (Yang et al., 2020). The data type processed has been expanded from a single spectral information to a combination of spectral data, spectral index and texture information, and the prediction accuracy and flexibility of the model have been further improved (Popescu et al., 2023).

## 5 Discussion

This paper reviewed the development and application of spectral remote sensing technology in the monitoring and inversion of nitrogen in fruit trees. It summarized and analyzed a large number of research results on the monitoring and inversion of nitrogen content in fruit trees. The importance of nitrogen for the development and growth of fruit trees has been fully confirmed. The feasibility of using spectral remote sensing technology to monitor and invert nitrogen in fruit trees has also been confirmed, providing valuable theoretical basis and technical support for further research and application.

It is very important to select the appropriate data acquisition method before the nitrogen monitoring and inversion process. With its excellent spatial analysis ability, remote sensing technology provides a large-area and continuous real-time non-destructive monitoring method for orchard nitrogen. Among them, the satellite remote sensing platform is limited by factors such as resolution, acquisition cycle and atmospheric environment, and is less used in orchards. On the contrary, it is widely used in the monitoring of field crops biomass. Firstly, the planting area of field crops is generally larger than that of orchards. The resolution of satellite spectral images can meet the research requirements of field crops non-details, and the long monitoring period is also in line with the growth trend of field crops. Airborne remote sensing platform, compared to satellite ones, offer higher image resolution and greater flexibility. They enable adjustments to flight altitude and select spectral cameras based on the characteristics of the research area. However, its promotion and application are constrained by economic factors; specifically, the costs associated with operating, maintaining, and managing aircraft are higher than those of UAV platforms. In contrast, UAV remote sensing platforms offer distinct advantages in terms of resolution, portability, and cost-effectiveness. So it has become one of the common methods for nitrogen monitoring in orchards and field crops. However, most UAV operations currently have relatively simple trajectories (straight lines or continuous fixed points) and require more sophisticated hardware to meet complex task requirements and working environments (Wang et al., 2023). It is worth noting that remote sensing methods mostly acquire image data at an angle perpendicular to the canopy, which means that the vertical distribution within the canopy has not been fully explored.

During the process of obtaining spectral data, both the parameter settings of the remote sensing platform and the spectral datasets influence the model inversion results. Taking the widely used UAV remote sensing platforms as an example, **Table 4** summarizes the parameter settings and dataset conditions. When setting flight parameters, altitude, speed, and image overlap rate are closely related to the study area, with each parameter influencing the others. If the flight altitude is too high, the resolution is lower. Flying too fast can lead to image blurring, while a high overlap rate results in excessive image repetition. Additionally, a low overlap rate can cause images to be inadequately stitched together. For the dataset of measured blades, an increase in data volume positively impacts the model’s accuracy. Furthermore, when using a single regression algorithm, models built with nonlinear algorithms tend to achieve higher accuracy than those using linear models. This difference is attributed to the existence of certain nonlinear relationships among various vegetation indices. On the other hand, multi-method ensembles are generally considered to yield more reliable results than the application of a single method alone (Feilhauer et al., 2015). It is worth exploring different ensemble methods and integrating various data types. Zhang et al. (2023) proposed an effective method based on ground and space remote sensing data fusion for the inversion of apple orchard CNC. This method utilizes a convolutional computation spectral response function to integrate ground-based high-spectral and drone-acquired multispectral data (Zhang et al., 2023). Gutierrez et al. (2023) used deep learning technology combined with multi-sensor spectral fusion technology to simulate grape components. The statistical analysis results confirmed that the fusion architecture performed better than the single spectral range model, demonstrating the potential of fusion technology in agricultural spectroscopy (Gutierrez et al., 2023).

**Table 4:** Summary of parameter settings and spectral dataset situation for the UAV remote sensing platform.

UAV	Flight parameters.	Spectral type.	Dataset.	Conclusion	References
DJI M600 PRO with Cubert UHD 185-Firefly camera.	Flight altitude:50m Image overlap:80%	Spectral range: (450 –950) nm, 125 bands spaced at 4 nm	Hyperspectral and RGB images of the canopy, a total of 1200 leaf samples, training set: verification set ratio = 2:1	Among the nitrogen content retrieval models, the best model was the SVM model ( $R^{2}$ =0.73)	(Li et al., 2022a)
DJI M600 PRO with Cubert UHD 185-Firefly camera.	Flight altitude:50m Image overlap:80%	Spectral range: (450 –950) nm, 125 bands spaced at 4 nm	Hyperspectral and RGB images of the canopy, a total of 1104 leaf samples, training set: verification set ratio = 2:1	Among the canopy nitrogen content retrieval models, the best model was the BPNN model ( $R^{2}$ =0.77)	(Li et al., 2022b)
DJI Phantom 4	Flight altitude:70m Image overlap:70%	Blue: 450 nm, Green:560 nm, Red: 650 nm, Red-Edge: 730nm Near-infrared: 840 nm	Multispectral and RGB images of the canopy, a total of 4000 leaf samples, the training points consisted of 90% (80% train and 10% validation) of the entire data-set, while the testing points were represented by the remaining 10%	Among the canopy nitrogen content retrieval models, the best model was the BPNN model ( $R^{2}$ =0.77)	(Osco et al., 2019)
eBee enseFly with MicaSense RedEdge-M™ camera	Flight altitude:100m Image overlap:85%	Blue: 475 nm, Green:560 nm, Red: 668 nm, Red-Edge: 717nm Near-infrared: 840 nm	Multispectral and RGB images of the canopy, a total of 70 leaf samples, leaf samples were divided into a calibration dataset (n = 53) and a validation dataset (n = 17).	Among the nitrogen content retrieval models, the best model was the ANN model ( $R^{2}$ =0.63)	(Noguera et al., 2021)

$\;R^{2}$ is the coefficient of determination.

In terms of nitrogen inversion algorithms, with the deepening of research, the form of machine learning methods has also been expanded. In the process of constructing regression models, traditional algorithms such as PLSR, MLR, and SMR are introduced. The combined prediction model can further improve the estimation accuracy of nitrogen content (Fu et al., 2020a; Shahhosseini et al., 2020). Mahajan et al. (2021) utilized spectral remote sensing data and a combined approach of PLSR and machine learning models to monitor mango leaf biomass (Mahajan et al., 2021). In addition, the process of regression modeling requires a certain amount of measured data to improve the robustness of the model. However, in some cases, the quantity and quality of measured data may not meet the expected targets. In contrast, physical methods based on physical principles have made great contributions to nitrogen remote sensing monitoring. Through the canopy radiative transfer mode (RTM), the correlation between biomass characteristics and canopy reflectance is clarified. In theory, the model has good interpretability and mobility (Abdelbaki and Udelhoven, 2022). However, in practical applications, the setting of model parameters and the mastery of physical principles limit its wide application. Therefore, the study of hybrid models can better leverage the strengths of statistical analysis and physical analysis methods. From a methodological perspective, the main feature is the combination of RTM-simulated data with actual measured data, incorporating biomass information into the spectral data to construct a hybrid dataset for feature selection. From a modeling perspective, hybrid models enhance the dataset by incorporating RTM-simulated data into the subsequent data training process for modeling. Li et al. (2024) introduced a method that combines UAV hyperspectral data and RTM simulations for the quantitative estimation of nitrogen content in corn leaves and canopies. By integrating field measurement data with RTM model simulation data, they conducted a comparative analysis of the performance of the hybrid method and the GRS method under different dataset conditions. The results showed that the hybrid method performed best in predicting nitrogen content in leaves. This paper believes that the hybrid method has broader application prospects and research significance in the monitoring and inversion of nitrogen content in fruit trees, and it can be considered as a direction for further research.

To enhance the accuracy of nitrogen monitoring in fruit trees, this paper suggests that investigating the influence of phenological periods on nitrogen monitoring in fruit trees could be a direction for future research. Generally, LNC will show a phased change trend at different phenological periods of fruit trees (Hueso et al., 2021). During the new shoot growth stage, trees have a longer growing season and more time to accumulate nitrogen, resulting in higher leaf nitrogen content, which in turn lays the foundation for the growth and development of fruit trees and their fruits. During the flowering stage, as the fruit trees grow and the leaves continue to expand, it is necessary to maintain sufficient nitrogen levels for chlorophyll synthesis. However, the nitrogen balance must be maintained at this stage. Excessive nitrogen content will lead to uneven distribution of nitrogen between flowers and leaves. The nitrogen required for flowering and fruiting is continuously used for photosynthesis to promote the growth of new leaves. The nitrogen content of leaves at this stage is relatively low. During the fruit enlargement period, the nitrogen content in the leaves is further reduced to maintain the nutrients required for fruit development and quality. At fruit maturity stage, nitrogen absorption rate slows down, leaf nitrogen content is mainly used to promote fruit maturity and storage time, and nitrogen is gradually transported to other plant parts through senescent leaves (Iqbal et al., 2022; Zhang et al., 2022b; Zhao et al., 2022). Peng et al. (2022) utilized a six-rotor M600 UAV (SZ DJI Technology Inc., Shenzhen, Guangdong, China) equipped with a six-channel multispectral Micro-MCA camera (Tetracam Inc., Chatsworth, CA, US) to collect images. The correlation between VIs and LNC was analyzed at the new shoot growth stage, flowering stage, fruit expansion stage and fruit Maturity Stage of grapes. The results show that the correlation of various VIs in different periods was different (Peng et al., 2022). In addition, the difference of vegetation index at different growth stages is also related to fruit yield (Ferro et al., 2023). Therefore, incorporating the phenological period characteristics of fruit trees into the research and analysis of nitrogen monitoring holds certain significance.

## 6 Conclusions

The real-time and accurate monitoring of nitrogen content in fruit trees is crucial for improving fruit yield and quality, as well as for influencing the formulation of fertilization plans, and promoting soil, water resources, and environmental protection. Spectral remote sensing technology, with its non-destructive, high-resolution, and real-time characteristics, provides an effective and reliable method for monitoring nitrogen content in fruit trees. Based on relevant literature, this paper introduced a framework for monitoring and inversion of nitrogen content in fruit trees, which primarily comprised three components: (1) The utilization of spectral remote sensing technology in monitoring nitrogen in fruit trees. (2) Criteria for assessing nitrogen status in fruit trees. (3) Formulation of algorithms for nitrogen regression inversion.

Firstly, the application of different remote sensing platforms in nitrogen monitoring of fruit trees was illustrated. Among them, the UAV remote sensing platform has been widely used due to its excellent flexibility and operability. At the same time, the portable measuring instrument also provides a new idea for the acquisition of the original data set, which enriches the types of data sets. Secondly, the indexes used to reflect the nitrogen status were summarized from the perspectives of leaves, fruit tree canopy, water and soil, so as to promote the further research of multi-index fusion. Finally, the regression algorithm were classified and discussed. Based on the complexity and linear relationship of the data, the characteristics and applicability of the algorithm were analyzed from the perspectives of traditional regression method and machine learning method, which provides a reference for selecting the appropriate modeling method. In summary, there is still room for further development of the current orchard fine management level. In the future, further research should be carried out on the integration of multiple data types and the phenological period characteristics of fruit trees.
