# Supplementary material for: Nitrogen monitoring and inversion algorithms of fruit trees based on spectral remote sensing: a deep review
Source: Front Plant Sci. 2024 Nov 22;15:1489151. doi: 10.3389/fpls.2024.1489151 (PMC11648862; doi:10.3389/fpls.2024.1489151)
Supplement: Supplementary file 1 [file Table1.docx]

Supplementary Material

# Abbreviations

| Abbreviation | English Full Name | Abbreviation | English Full Name |
| --- | --- | --- | --- |
| Adaboost | Adaptive boosting | NDRE | Normalized difference red-edge |
| ANN | Artificial neural network | NDVI | Normalized difference vegetation index |
| BPNN | Back propagation neural network | NDWI | Normalized difference water index |
| CCCI | Canopy chlorophyll content index | PLSR | Partial least squares regression |
| CNC | Canopy nitrogen content | PolSAR | Polarimetric synthetic aperture radar |
| CNN | Convolutional neural network | RBF | Radial basis function |
| GBDT | Gradient boosting decision tree | RDVI | Renormalized difference vegetation index |
| GBM | Gradient boosting machine | RTM | Radiative transfer model |
| GPR | Gaussian process regression | RF | Random forest |
| LightGBM | Light gradient boosting machine | SAVI | Soil-adjusted vegetation index |
| LMC | Leaf moisture content | SMR | Stepwise multiple regression |
| LNC | Leaf nitrogen content | SVM | Support vector machine |
| LSR | Least square regression | SVR | Support vector regression |
| MLP | Multilayer perceptron | TSAVI | Transformed soil-adjusted vegetation index |
| MLR | Multiple linear regression | UAV | Unmanned aerial vehicles |
| MSAVI | Modified soil-adjusted vegetation index | VIs | Vegetation indices |
| NDCSI | Normalized difference canopy shadow index | XGBoost | Extreme gradient boosting |
| NDNI | Normalized difference nitrogen index |  |  |
